# Patients With Cancer in the Countries of South-East Europe (the Balkans) Region and Prospective of the Particle Therapy Center: South-East European International Institute for Sustainable Technologies (SEEIIST)

**DOI:** 10.1016/j.adro.2021.100772

**Published:** 2021-08-09

**Authors:** Mimoza M. Ristova, Vesna Gershan, Herwig Schopper, Ugo Amaldi, Manjit Dosanjh

**Affiliations:** a Faculty of Natural Sciences and Mathematics, Physics Department, University Ss Cyril and Methodius in Skopje, North Macedonia; b SEEIIST Association, Geneva, Switzerland; c CERN, Geneva, Switzerland; d TERA Foundation, Novara, Italy; e Department of Physics, University of Oxford, Oxford, United Kingdom

## Abstract

**Purpose:**

A recent initiative was launched for establishing the South-East European International Institute for Sustainable Technologies (SEEIIST), which will provide a cutting-edge Hadron radiation therapy treatment and research institute for treating cancer patients with Hadron therapy (HT). To justify the initiative for building the SEEIIST facility, a study was conducted to estimate the number of patients with cancer from the SEE region that would be eligible for HT.

**Methods and Materials:**

Two different methods for projecting the future annual cancer incidence have been applied: (1) using the International Agency on Research on Cancer@World Health Organization's (WHO) Globocan model which uses country's demographic factors, and (2) averaging the crude incidence data of 3 SEE countries with available national cancer registries, using a linear regression model of combined incidence per 100,000, and applying it to the entire SEE region. Cancer epidemiology data were collected and studied by using the countries’ cancer datasheets from WHO. The top 10 cancers were presented for the SEE region. Studies of other countries were used to develop a primordial model for estimating the number of SEE patients who could be treated most successfully with HT upon SEEIIST commissioning in 2030.

**Results:**

A model was developed to estimate the number of eligible patients for HT from SEE. It is estimated that 2900 to 3200 patients per year would be eligible for HT in the new SEEIIST facility in 2030.

**Conclusions:**

After commissioning, SEEIIST will initially treat approximately 400 patients per year, progressing toward 1000. Creation of SEEIIST dedicated patient selection criteria will be both necessary and highly challenging.

## Introduction

Cancer is the second leading cause of death globally. The number of new patients with cancer in the world is in constant growth. According to the World Health Organization (WHO), the number of new cases in 2018 was 18.1 million: 9.6 million people died, and 43.8 million people are living with cancer.^1^ It has to be noted that approximately 70% of cancer deaths come from low- and middle-income countries.^2^ The battle with cancer is most challenging in the development of cancer therapy modalities for difficult to treat cases, such as childhood, deep-seated, and radioresistant cancers. Demographic drivers of increasing world population, aging populations (particularly in higher-income countries), along with the progress in the reduction of many other causes of deaths, implies that the total number of cancer deaths will continue to increase.^3^ It is also evident that about 50% of patients with cancer benefit from radiation therapy (RT).^4^ European epidemiologic studies related to the Italian,^5^ Austrian,^6^ and French,^7^ Hadron therapy (HT), also called particle therapy, centers, carried out under the European Network for Light Ion Hadron Therapy Network,^8^ made it possible to establish a consensus of priority cases for this type of therapy. Most of the eligible patients with cancer have difficult-to-treat cancers: radioresistant, inaccessible sites, deep-seated, or situated next to radiosensitive organs. Recent clinical studies have proven that HT became treatment of choice for a number of specific types of cancers.^9^^,^^10^ For several cancer types, such as skull base chordomas, the 5 or 10-year survival rate with HT is 2 to 3 times higher than the best results obtained with conventional (x-ray) RT.^11^ HT is the best choice of treatment for a number of pediatric cancers,^12^ locally advanced esophageal cancer,^13^ as well as a range of other cancers. Tumors eligible with the highest priority for proton therapy include adults’ skull base, unresectable or relapsing meningioma, and rare adults’ central nervous system tumors. Types of tumors eligible with highest priority for ion therapy (carbon) include adenoid cystic carcinomas of salivary glands, including head and neck and thorax, sinus adenocarcinomas, chordomas, and chondrosarcomas of skull base and spine, soft tissues sarcomas, and non-small cell lung carcinomas as well as pelvic local relapses of adenocarcinomas.^14^^,^^15^

South-East European (SEE) countries are in the Balkan Peninsula (Appendix E1), with a population of about 43 million: Albania (3 million), Bosnia and Herzegovina (3.8 million), Bulgaria (7.1 million), Croatia (4.3 million), Greece (10.7 million), Kosovo (1.9 million; in this article, the designation to Kosovo is without prejudice to positions on status and is in line with United Nations Security Council 1244/1999 and the International Court of Justice opinion on the Kosovo Declaration), Montenegro (0.6 million), North Macedonia (2.1 million), Serbia (7.1 million), and Slovenia (2.1 million). Besides the geographic meaning, they share some common historical, political, and societal challenges. Four out of 10 SEE countries (Bulgaria, Croatia, Greece, and Slovenia) are part of the EU, and the other 6 are associated members. Another common phenomenon in the SEE countries is high emigration rates, resulting in a substantial decrease in their population.^16^ According to the World Bank,^17^ 7 out of 10 SEE countries are middle-income countries. Hence, they are facing a greater number of challenges in combating cancer compared with Western Europe, which is due to the lack of national and regional strategies, organized reliable screening programs for early cancer detection, campaigns for preventing cancer, adequate state-of-the-art diagnostic, and treatment equipment, advanced treatment modalities, and cancer registries that can serve as a base for data analysis and strategic planning. South-East European International Institute for Sustainable Technologies (SEEIIST)^18^ is an Accelerator-based Research Infrastructure for Cancer Therapy and Biomedical Research with ion beams that will provide HT to the citizens of the SEE region who currently have no such facility. SEEIIST will also contribute to transferring the positive values of science into SEE politics and diplomacy, mitigating the historical friction among some SEE countries by after the positive experience of the European Council for Nuclear Research,^19^ which was established in 1954 at Geneva to provide a common language and purpose to scientists from different regions and promote cutting-edge physics research. It provided an infrastructure for peaceful collaboration to scientists and engineers from countries that were at war less than a decade before, resulting in the establishment of the most successful particle physics laboratory in the world. It has become a model for what is possible when people collaborate and was the template for SESAME, the “Synchrotron-Light for the Experimental Science and Applications in the Middle East,” which was launched under the auspices of United Nations Educational, Scientific and Cultural Organization and officially opened in Allan, Jordan in 2017, with 8 member states, including Iran and Israel for peaceful and cutting-edge collaboration. Both European Council for Nuclear Research and SESAME were influential for the SEEIIST foundation. SEEIIST facility is currently in the second phase of technology development and defining the criteria for site selection within the SEE region. SEEIIST is expected to be fully commissioned in 2029 to 2030.^20^ The number of patients to be initially treated at SEEIIST will be about 400 per year (both with protons and C-ions), progressively growing to more than 1000, as soon as the facility is fully optimized. To ensure the gathering of systematic regional cancer epidemiologic data in the region, as well as a comprehensive analysis of the incidence and types of cancers there is a need to: (1) establish a regional cancer scenario for SEE; (2) estimate/project the number patients who will need RT, and in particular those who will benefit the most from HT; (3) demonstrate the need for the SEEIIST facility for patients of the SEE region, and (4) develop selection criteria for patients to match the SEEIIST treating capacity.

Herein, we present the results of our analysis of the available data on cancer patients in the SEE region, including cancer incidence, incidence-to-mortality rates, epidemiology, and projections for the future decades based on (1) the currently available Globocan (GC) tool, which uses the projections of the demographic trends and the economic factors before the COVID-19 pandemic and Brexit, and (2) public available national cancer statistics (NS) for Bulgaria, Croatia and Slovenia that were collected by WHO (CI5,^4^ Cancer Incidence in 5 Continents).

This research is useful for SEE countries’ cancer treatment stakeholders in their timely strategic planning and budgeting for the treatment of their cancer patients in the decades to come. Moreover, this research should serve as a wake-up call that could bring the SEE region together in a common alliance against their common enemy: cancer. The ultimate ambition of this research is to increase the awareness of the influential visionaries among the SEE health care stakeholders, to catalyze them to seize the window of opportunity and enable the HT facility-SEEIIST to become a reality.^20^^,^^21^

Some of the reported estimates^22^ that predict up to 15% to 20% of all the RT patients would be eligible for HT was probably too optimistic. Currently, a more realistic view is that approximately 2% and 5% of RT patients would be eligible for HT in 5 and 10 years, respectively, mainly confined to the geographic areas in which HT currently is or will be available in the upcoming years. Despite this low percentage of patients suitable for HT, only in Europe (740 million of population), the potential need for HT is expected to grow from 40,000 to 100,000 (ie, multiple times the capacity of the current and planned HT centers). In addition to this, and the recognized clinical evidence for the benefit from HT, it became clear that the need for HT should be evaluated on an individual basis, which is an achievable task. Alternatively, novel models for HT patient selection could also be applied to offer particle therapy to the patients who can benefit the most from this modality. A Dutch group^23^ proposed a model-based approach consisting of 2 phases. The selection in the first phase is based on the patient-populations that will most likely benefit from HT by using normal tissue complication probability value approach. In the second phase such results are clinically validated using sequential prospective observational cohort studies of appropriate historical comparisons as a reference.

## Methods and Materials

Analysis, calculations, and projections are crucial for estimating the future cancer burden in countries up to 2040. GC, a database in the Global Cancer Observatory from the International Agency on Research on Cancer (IARC)@WHO,^24^ is a valuable tool for cancer incidence projections^25^ and the strategic planning of each country's burden. Also, as already noted in GC, the quality and coverage of cancer data worldwide remain limited from low- and middle-income countries, comprising the majority in the SEE region. Furthermore, the IARC^26^ stated that not only is it important to evaluate, compile, and use the data from the Agency's cancer registry collaborators in projections for the future, but also to work alongside the national staff of each country to improve the quality of local data, local tumor registry coverage, and their capability to analyze the cancer data.

It is also important to mention that GC cancer predictions for future should be interpreted with due caution.^27^ Presently, it has become evident that projected numbers were based on the key assumption that the national migration rates have a known regular behavior. However, the 2020 to 2030 period brings considerable uncertainty and challenges in the prediction. First, there is an uncertainty related to the global and persistent SARS-CoV-2 pandemic, and measures, which are changing daily, are affecting the demography and migration of the SEE population and causing economic consequences. Second, reversible migrations due to the post-Brexit situation in Great Britain are also possible, causing additional economic effects.^28^ In other words, the latter could reverse the migration back to SEE inducing greater uncertainty of predicting future cancer behavior.

Our research encountered a challenge upon finding that the ten collaborating SEEIIST countries are only partly covered by reliable cancer data. There are ongoing discussions about creating publicly available national and regional cancer registries that would provide relatively good quality regional cancer epidemiology data, but the activities are still in planning. The current cancer country profiles at the WHO and the cancer data sets are the available sources for SEE regional projections for the decades to come. The oldest registry available is the one in Slovenia that covers the period from 1956 until 2012. The Croatian and Bulgarian national registries cover periods from 1988 to 2012 and 1998 to 2012, respectively. Also, there is a registry of Central Serbia that partly covers the country for a short period (1999-2002) and is therefore incomparable with the other 3. Hence, the available NS from the 3 SEE countries (Slovenia, Croatia, and Bulgaria) were used for projections of the cancer incidence in the entire SEE region because the data sets for these countries were assessed by GC to be of relatively good quality. The prediction for the cancer incidence in these countries in the present and future decades can be made by using a linear regression model of the crude incidence rate. A combined crude incidence from 3 SEE countries was calculated for 2020, 2030, and 2040. This crude incidence rate was used as a common SEE figure for future assessments for the countries with missing cancer data because the methodology for compensating the missing empirical data has been widely used by WHO and in many relevant cancer analysis publications.^29^^,^^30^ The projections for 2020, 2030, and 2040 were done under the assumption that the population in all 10 SEE countries will not change significantly in future decades. To the best of our knowledge, there are no studies that address cancer epidemiology in the SEE countries nor the eligibility of patients with cancer who would benefit the most from the hadron therapy (protons and C-ions). Numerous studies were made by other countries that estimate the incidence of the cancers per 100,000 population that would be best treated with HT. The studies reported were carried out independently in individual countries and vary in methodology so are not directly comparable. The average values of estimates based on the clinical experience of the functional HT facilities can be used to calculate the number of HT eligible patients in the SEE region to be treated in the SEEIIST facility, starting in 2030.

## Results

### Patients with cancer in the SEE region

#### Cancer incidence and mortality-to-incidence ratio in SEE region using GC model

**Fig. 1:**
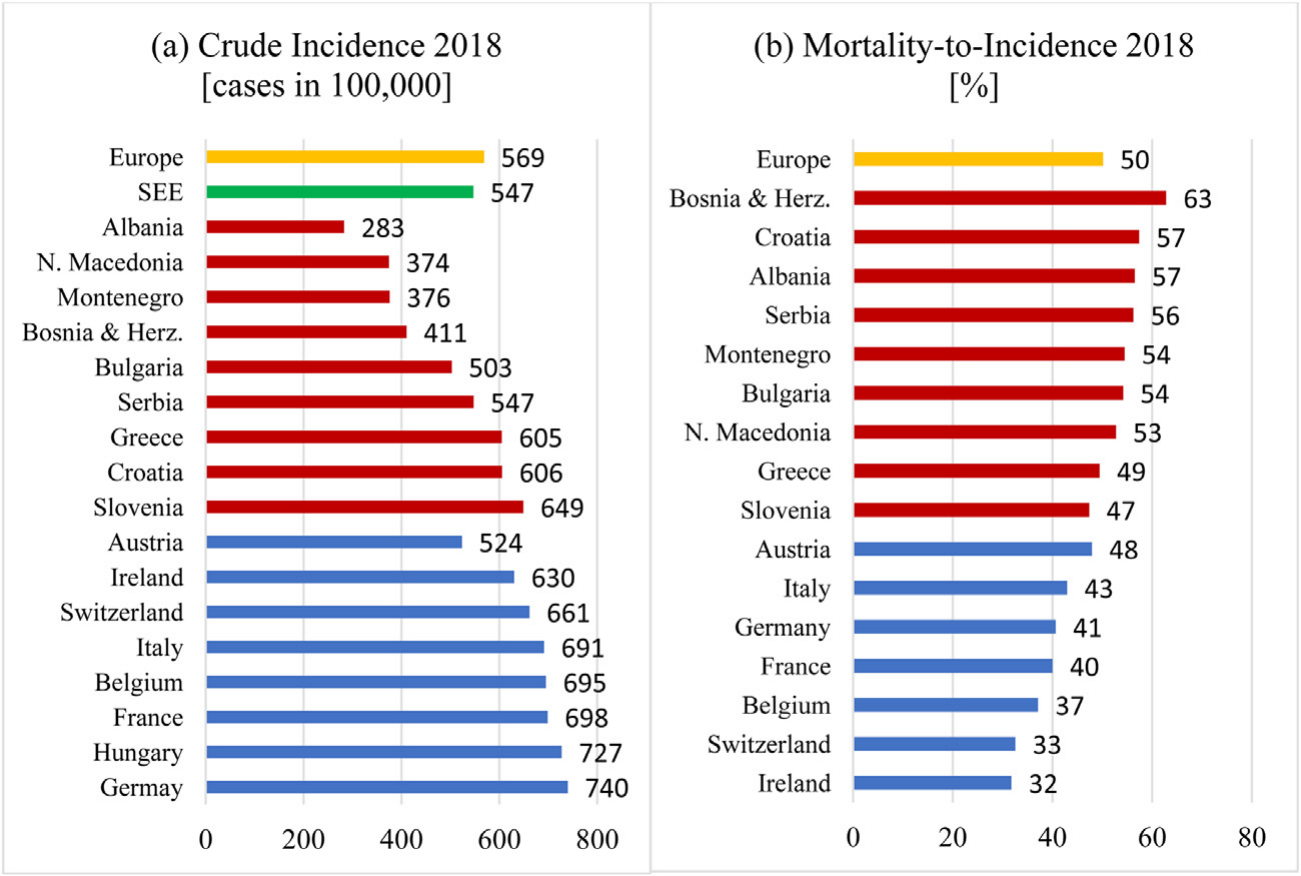
(a) Cancer incidence (crude rate per 100,000) in South-East European countries (except Kosovo) in comparison with some European countries. (b) Mortality-to-incidence ratio 2018, for all cancer cases except nonmelanoma skin cancers in the South-East European countries (except for Kosovo) red bars and several Western European countries for comparison (source: Globocan). *Abbreviations:* SEE = South-East European.

The incidence of new cancer cases (all cancers, both sexes, all ages) was estimated from the most recent available data (2018) made by IARC through collaborations with population-based cancer registries (the International Association of Cancer Registries) and with the World Health Organization or are based on information that is publicly available online. In addition, several Western EU countries have been selected for comparison with SEE. According to Figure 1a, the crude cancer incidence in 6 out of the 9 SEE countries presented falls below the European average. On the other side, all the selected EU countries, except for Austria, have cancer incidences above the European average. The highest crude rates are evident for Germany and Hungary. Furthermore, some of the SEE countries (Albania, N. Macedonia, Bosnia and Herzegovina, and Montenegro) have incidence rates about half of those in Germany and Hungary.

Another asset parameter is the mortality-to-incidence ratio (MIR), which is an important indicator, calculated by dividing the mortality rate by the incidence rate for a selected country for the same year. MIR is an indicator opposite to patient survival rate, and it points to the percentage of cancers with a fatal outcome. MIR indicates how efficiently and successfully cancer has been managed in different countries. The presented data in Figure 1b indicate that MIR in SEE is much higher compared with the most Central and Western European countries. Furthermore, MIR for 7 out of 9 SEE countries (data are missing for Kosovo) is above the European average, suggesting that cancer in the SEE region is less successfully managed by the health care systems. The situation is alarming for Bosnia and Herzegovina, for which the MIR is twice as large as those of Switzerland and Ireland.

### Top 5 cancers in the SEE region in 2018

Cancer epidemiology in the SEE region as a whole could be described with the top 5 cancers that occur with the highest incidence in the SEE region, calculated as a sum of the countries’ estimated age-standardized (World) incidence in 2018. According to the available Eurostat data, the top 5 SEE countries’ lists of cancers that require radiation therapy differ among each other (Appendix E2). The calculations showed that the top 5 cancer sites in the SEE region as a whole are (1) lung, (2) colorectum, (3) breast, (4) prostate, and (5) bladder, comprising more than 50% of the overall cancer incidence in 2018. For comparison, the global cancer epidemiology in 2012 shows different ranging: (1) breast, (2) lung, (3) prostate, (4) head and neck, and (5) colorectum.^29^ From these projections, it appeared that approximately 14% of all patients with cancer have lung cancers, making it the number 1 cancer in the SEE region as a whole, although on the national lists, it appears as number 2 in Croatia and Montenegro and number 3 in Bulgaria and Slovenia.

### Cancer patient projections for SEE

#### Projections for new cancer cases in SEE region using available NS

**Fig. 2:**
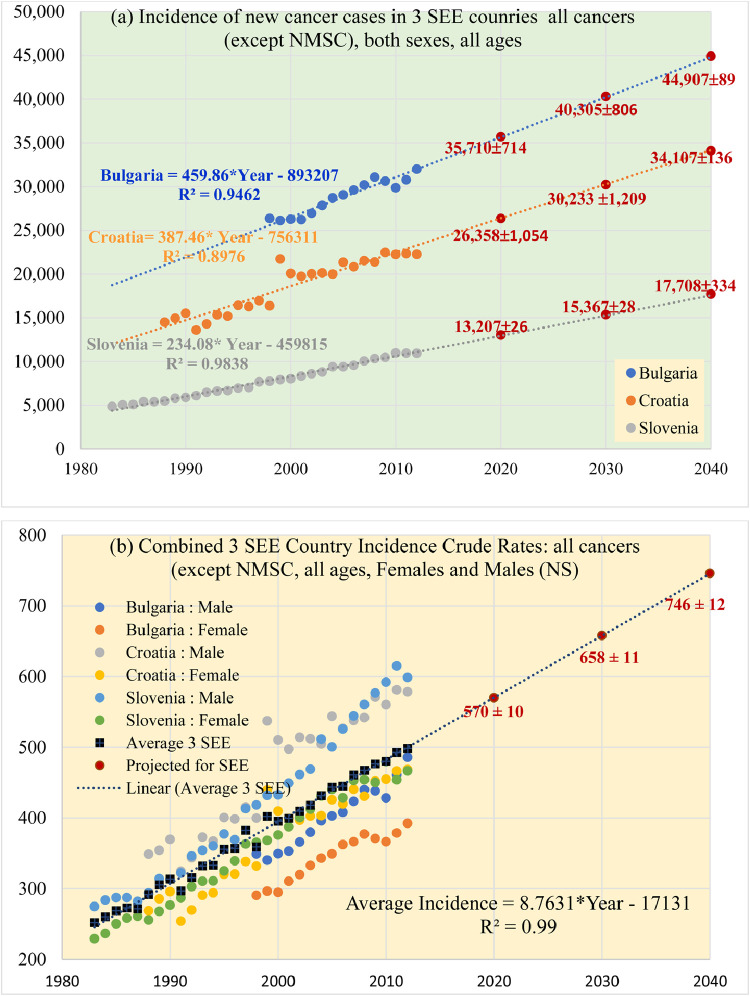
(a) New cancer cases in 3 South-East European (SEE) countries and projections for 2020, 2030, and 2040. All cancers except nonmelanoma skin cancers (all aged 0-85). The projected number of new cases is presented with red data marks and absolute numbers with the evaluated standard error (2% for Bulgaria, 4% for Croatia, and 2% for Slovenia). (b) Crude rates, all cancers except nonmelanoma skin cancers (all aged 0-85+). Note: Combined crude rate on 100,000 for the 3 SEE countries, was calculated as an average (black squares) of all the available data from each of the 3 countries for males and females. The projected combined crude incidence from the 3 SEE countries was calculated from the linear regression model for 2020, 2030, and 2040 (brown circles) along with the combined standard deviation of 2%. *Abbreviations:* NMSC = nonmelanoma skin cancers; SEE = South-East European.

The publicly available data for Bulgaria, Croatia, and Slovenia were in a form of an age-standardized (World) rate of all cancers except nonmelanoma skin cancers (NMSC). These data were used for analyzing the time progression of the new cancer patients in the 3 countries, as shown in Figure 2a. The projected numbers of new patients along with the standard errors for 2020, 2030, and 2040 were determined from the linear regression model for each of the 3 SEE countries.

The obvious growth of the new cancer cases from Figure 2a is a global phenomenon that has been explained, among other factors, by the aging of the world population and improvements in health care.^31^^,^^34^ The older the population, the higher the probability of getting cancer once in a lifetime. However, the cancer incidence in the population of children is not related to their “old age.” It could be correlated to the relevant risk factors, such as the genetics, quality of air, water, food, to available medical procedures for early detection, but also a preconception or prenatal radiation exposure to x-rays in diagnostic procedures.^32^^,^^35^

**Fig. 3:**
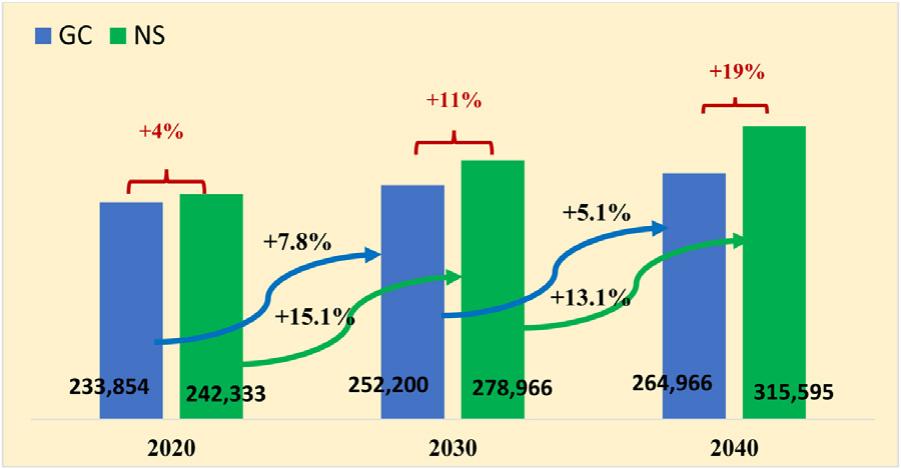
Projections of new cancer cases in the South-East European region in the future decades: Comparison of 2 different projection models: (1) Globocan data evaluation methodology (blue bars), and (2) national statistics linear regression model developed from empirical crude incidence of Bulgaria, Croatia, and Slovenia, then applied to the rest of the 7 South-East European countries. (Note 1: national statistics data are taken from Table 1. Note 2: Predictions for Kosovo are unavailable in Globocan source). *Abbreviations:* GC = Globocan; NS = national statistics.

Figure 2a shows the incidence (crude rate per 100,000 population, all cancers, except NMSC, for males and females separately, all ages (0-85+) for Bulgaria, Croatia, and Slovenia. The combined crude rate of incidence for the 3 SEE countries, calculated as a mean of the 3 SEE countries, as presented with black squares in Figure 2b, from which it is clear that the crude incidence also grows with time according to a linear model. However, this linear growth may be soon affected by the COVID-19 pandemic and Brexit. Finally, a dashed trend line is obtained from the linear regression model of the datapoints of combined crude incidence. The recurrent formula of the fit-line was used to predict the incidence of cancer for the SEE region as a whole for 2020, 2030, and 2040.

Table 1 presents a numerical summary of the predicted incidence using the NS, based on the combined crude incidence of 3 SEE countries from Figure 2b, calculated for the remaining 7 SEE countries for which national registry data were not available. Furthermore, the time evolution of the combined crude incidence per 100,000 population was applied to calculate the projected incidences (in absolute numbers) for each of these 7 SEE countries, simply by multiplying the average crude incidence with their current population in millions, multiplied by 10. Similarly, we calculated the incidence in the SEE region, as a whole. The projections for 2020, 2030, and 2040 were done under the assumption that the population in all SEE countries will not change in future decades.

**Table 1:** Projections of cancer incidence

A country with publicly available cancer registry data (NS)	Population 2020 (million)	NS: extrapolated incidence (data from Fig. 1, Fig. 2a)			GC-Projected incidence		
		2020	2030	2040	2020	2030	2040
Bulgaria	7.05	35,710	40,309	44,907	35,366	35,258	34,504
Croatia	4.27	26,358	30,233	34,104	25,454	26,851	27,845
Slovenia	2.08	13,027	15,367	17,708	13,934	16,004	17,417
Combined 3 SEE crude incidence on 100,000 (from Fig. 1b)		570	658	746			
A country with no empirical data		NS Projected incidence using combined 3 SEE crude rates					
Kosovo*	1.91	10,887				9,223	10,052
Montenegro	0.61	3,477	4,014	4,551	2,423	2,704	2,888
N. Macedonia	2.08	11,856	13,686	15,517	8,048	9223	10,052
Serbia	7.08	40,356	46,586	52,817	48,471	49,682	49,273
Albania	3.05	17,385	20,069	22,753	8,604	10,049	11,197
Bosnia and Herzegovina	3.85	21,945	25,333	28,721	14,771	16,638	17,231
Greece	10.76	61,332	70,801	80,270	68,725	76,568	84,507
Total SEE	42.74	242,333	278,966	315,595	233,854	252,200	264,966
Increase relative to the previous decade			+15%	+13%		+7.8%	+5.1%

*Abbreviations*: GC = Globocan; NS =national cancer statistics; SEE = South-East European.

Projections are calculated through (1) NS: available national statistics for Bulgaria, Croatia, and Slovenia, and the 3 SEE country combined crude incidence evaluated with linear regression from Figure 2b. The incidence in Albania, Bosnia and Hercegovina, Greece, Kosovo, Montenegro, North Macedonia, and Serbia was projected from the combined 3 SEE country crude incidence; (2) GC: projected incidences by Globocan. Herein, projections for Kosovo are not available.

Therefore, the data blanks for Kosovo are filled in by copying the Globocan data for North Macedonia.

⁎ Unavailable data for Kosovo. Data in the table are duplication of the Globocan data for North Macedonia.

#### Projections for new cancer cases in the SEE region using GC

Herein, GC predictions for future cancer incidence in the SEE countries are also presented in Table 1. As can be seen, all the countries show a growth in cancer incidence, starting from 2020 to the future decades (2030 and 2040) except for Bulgaria, where a decline in the incidence is projected for the future. This trend remains a mystery that is unlikely to hold. Also, data for Kosovo is missing in the GC application. For the sake of comparison of the 2 models NS and GC, we have speculated that the GC projected incidence for Kosovo could be equal/close to that of North Macedonia.

#### Comparison of the 2 projection models (GC and NS) for new cancer cases in the SEE region

Figure 2 depicts the projected cancer incidence in the SEE region for 2020, 2030, and 2040. The 2 projections are based on (1) NS data sets from Bulgaria, Croatia, and Slovenia, shown in Table 2, and (2) GC projections, both for the cancer incidence in the SEE region. There is a notable difference between the NS and the GC incidence projection. Herein, from Table 1 it is evident that GC and NS projections are in relatively good agreement for Slovenia. For other SEE countries the 2 models yield slightly differing results for the future. The discrepancy between the 2 models for Bulgaria and Croatia is substantial for future decades. For Albania, Montenegro, North Macedonia, and Bosnia and Herzegovina the discrepancies for 2030 are greater than 30%. The latter could be a result, among others, of the higher projected immigration rates in these 2 countries in the GC model. As we noted earlier, the COVID-19 pandemic and Brexit are expected to effect the demography dynamics and slow down the emigration from the SEE region and hence, GC cancer figures will diverge from reality.

**Table 2:** A list of studies for patient selection eligible for HT and their crude incidence in different countries

No.	Country	New cancer cases and RT candidates	Incidence of candidates for PT on 100,000	Incidence of candidates for p^+^ on 100,000	Incidence of candidates for C-ions on 100,000
1	Austria (6)	New = 36,500 RT = 15,132 (41%)	HT = 13.5% of RT	HT = 2044 (all Austria) population (2004) = 8.2 million 25 cases on 100,000	
2	Italy (5)	25,000 new radioresistant tumors	10%-15% of radioresistant = 3000-4000 cases	Population 57.5 million population (2005) 6.9 cases on 100,000	
3	Iran (29)	2,932 cases 1,943 for RT 66% of cancer cases	HT = 22.9% of RT	5,756 eligible for HT, entire Iran Population 78.5 million population (2018) 7.3 cases on 100,000	
4	France (4)	One day study (5 clinics); survey on 532 RT candidates	HT =77/532 RT HT= 14.5% of RT	5.320 eligible for HT, entire France Population 65.5 million population (2013) 8.5 cases on 100,000	
5	France (5)		HT = 12% of RT	7 cases on 100,000	5 cases on 100,000
				7500 eligible for HT, entire France Population 64 million population (2006) 12 cases on 100,000	
5	Sweden (20)	31,050 cases 7,650 for RT 25% of cancer cases	HT = 15% of RT	2,475 eligible for HT, all Sweden Population (2005) = 9 million 28 cases on 100,000	
6	Yellow report (7)			First priority protons: 0.8 cases on 100,000	First priority C-ions: 2 cases on 100,000
				Second priority: 5 cases on 100,000 I and II priority: 7.8 cases on 100,000	
7	S. Korea (8)			4000 eligible for C-ions, all S. Korea Population (2018) = 51.6 million 7.8 cases on 100,000	

*Abbreviations*: HT = Hadron therapy; PT = particle therapy; RT = radiation therapy.

Furthermore, the effects of the COVID-19 pronounced mortality rates for the older population could further affect the age structure of the SEE population. Many unknown developments make the new cancer case projections inaccurate. It follows that the future strategies for combating cancer should be based on the more pessimistic NS-model, because the GS-model may be too optimistic and grossly underestimate the cancer incidence by about 11% for 2030 (Fig. 2).

#### Cancer patients from the SEE region eligible for HT

##### Projections of particle therapy patients from SEE using the average crude incidence of eligible patients

If we apply the calculated average incidence (adopted in Section 2) of 7.8 new patients with cancer per 100,000 population on the present SEE population of about 43 million (2020), we can estimate that the absolute number of new patients with cancer eligible for HT is 3311. As was shown in Figure 1a, the crude cancer incidence in the countries that possess HT centers (such as Italy, France, Germany, Switzerland) is about 700 and in the SEE region is about 550 (about 20% less). Hence, one should consider downgrading roughly the 7.8 crude incidence by 20%. From here it follows that the number of new patients with cancer *P*(SEE), calculated from the incidence of the HT eligible patients in 2020, will be estimated to

(Equation 1) P(SEE)=80%·7.8·42.7Millions·10=2687patients.

**Fig. 4:**
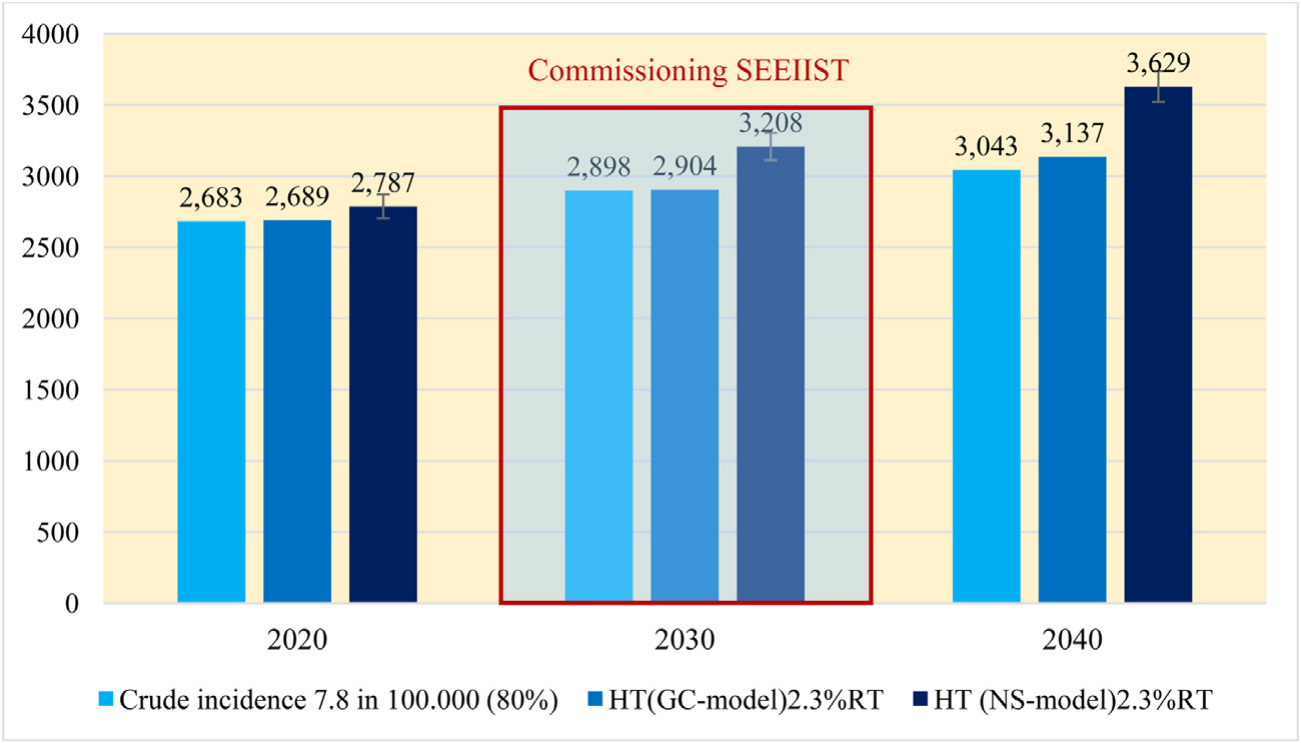
Incidence of eligible patients in from South-East European for hadron therapy in 2020, 2030 and 2040 (all ages, all cancers) according to Globocan and national statistics (under the assumption that the population remains 43 million until 2040). *Abbreviations:* GC = Globocan; HT = hadron therapy; NS = national statistics; RT = radiation therapy; SEEIIST = South-East European International Institute for Sustainable Technologies.

By applying the projected growth rates, one can estimate that the number of the new patients with cancer who can benefit most from HT be as follows: 2687, 2898, and 3043 for 2020, 2030, and 2040, respectively, as presented with the light blue bars in Figure 4.

#### Projections using the cancer incidence in the SEE region as a parameter

According to the more optimistic GC scenario, the total number of patients with cancer from the SEE region in 2020, 2030, and 2040 is estimated to 233,845, 252,200, and 265,000, respectively. Assuming that 50% of those patients will be referred to receive RT as a part of their treatment, we can estimate that 116,922, 126,100, and 132,500 patients in 2020, 2030, and 2040, respectively will be referred for RT. Using the more pessimistic scenario (NS incidence data) we have calculated that e number of the new RT patients comprising 50% of the previous to be 121,166, 139,483, and 157,797 for 2020, 2030, and 2040 new RT patients, respectively.

As it was presented in Figure 3a, the average crude incidence in the SEE countries is rather lower than that of the Western European countries. Also, it appeared that the cancer epidemiology of the SEE region differs from that of the European average, as was shown in the top 10 list of cancers (Table 1). Because the SEE region reveals regional characteristics: (1) the cancer incidence is lower than that in Western Europe, (2) the mortality-to-incidence ratio is higher than that of Western Europe, and (3) last but not least, due to the specific cancer epidemiology, for the sake of future estimations it will be convenient to recalibrate the incidence of the HT eligible cancer patients (Equation 1) as a percentage of RT patients. Herein, for 2020 the calculation gives that those 2687 patients would be eligible for HT represents 2.3% of the RT patients (50% of the new patients with cancer, all cancers, all ages). Now, once we adopt the HT eligibility criterion of 2.3% of the RT patients, we can apply it to the 2 predictive models of cancer incidences, GC and NS. The number of cancer patients from SEE who will qualify for HT in 2030 could be estimated to be 2900 (GC) to 3200 (NS), as depicted in Figure 4.

## Conclusions

Assuming that the SEEIIST facility will be commissioned for patient treatment in 2029 to 2030 and that its initial treatment capacity^20^ will be about 400 patients per year, progressing toward 1000, it appears that the creation of patient selection criteria will pose a complex challenge. On its commissioning in 2030, SEEIIST shall select 400 patients of 2900 to 3200 HT eligible patients, calculated as 2.3% of the RT patients. From here it follows that the countries of the SEE region should initially develop a 2-steps qualification criterion, similar to the so-called model-based selection.^33^^,^^36^

The wide range of the incidence of patients who would be candidates for HT in 2030, reported by different studies, indicates a global lack of standardized selection criteria. To develop SEEIIST regional standardized selection criteria, it is a prerequisite to have functional national cancer registry in each country within the SEE region, as the first step in establishing a SEE regional cancer database. In parallel, it will be necessary to upgrade the national strategies for early-stage cancer diagnosis and treatment in the SEE countries.

## Appendix Supplementary materials

Image, application 1

## Footnotes

Supplementary material associated with this article can be found, in the online version, at doi:10.1016/j.adro.2021.100772.
