# Supplementary material for: Patients With Cancer in the Countries of South-East Europe (the Balkans) Region and Prospective of the Particle Therapy Center: South-East European International Institute for Sustainable Technologies (SEEIIST)
Source: Adv Radiat Oncol. 2021 Aug 9;6(6):100772. doi: 10.1016/j.adro.2021.100772 (PMC8581504; doi:10.1016/j.adro.2021.100772)
Supplement: Supplementary file 1 [file mmc1.docx]

**ANNEX 1**

Map of the ten SEE countries on the Balkan peninsula and their population (total of 43 millions)


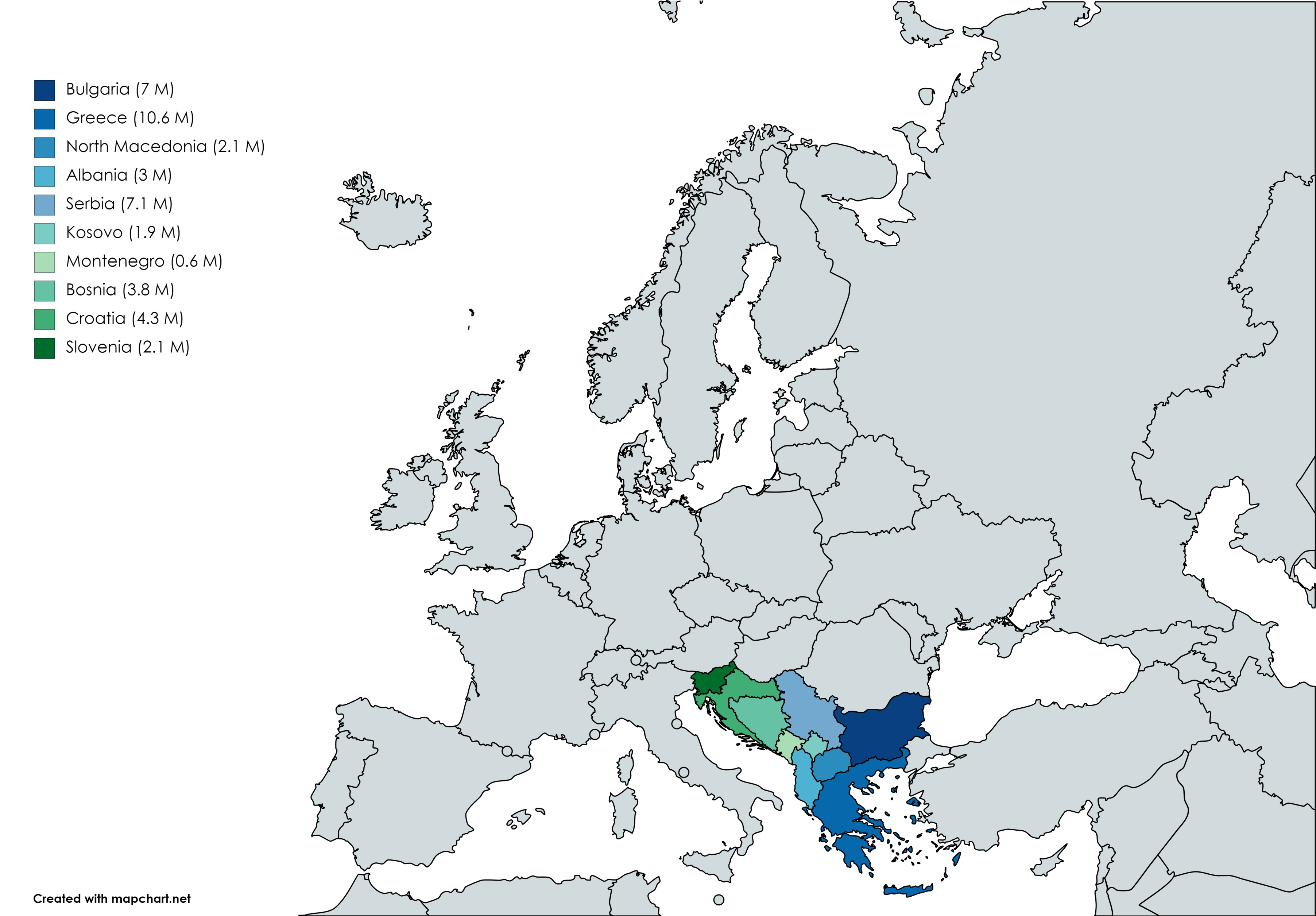

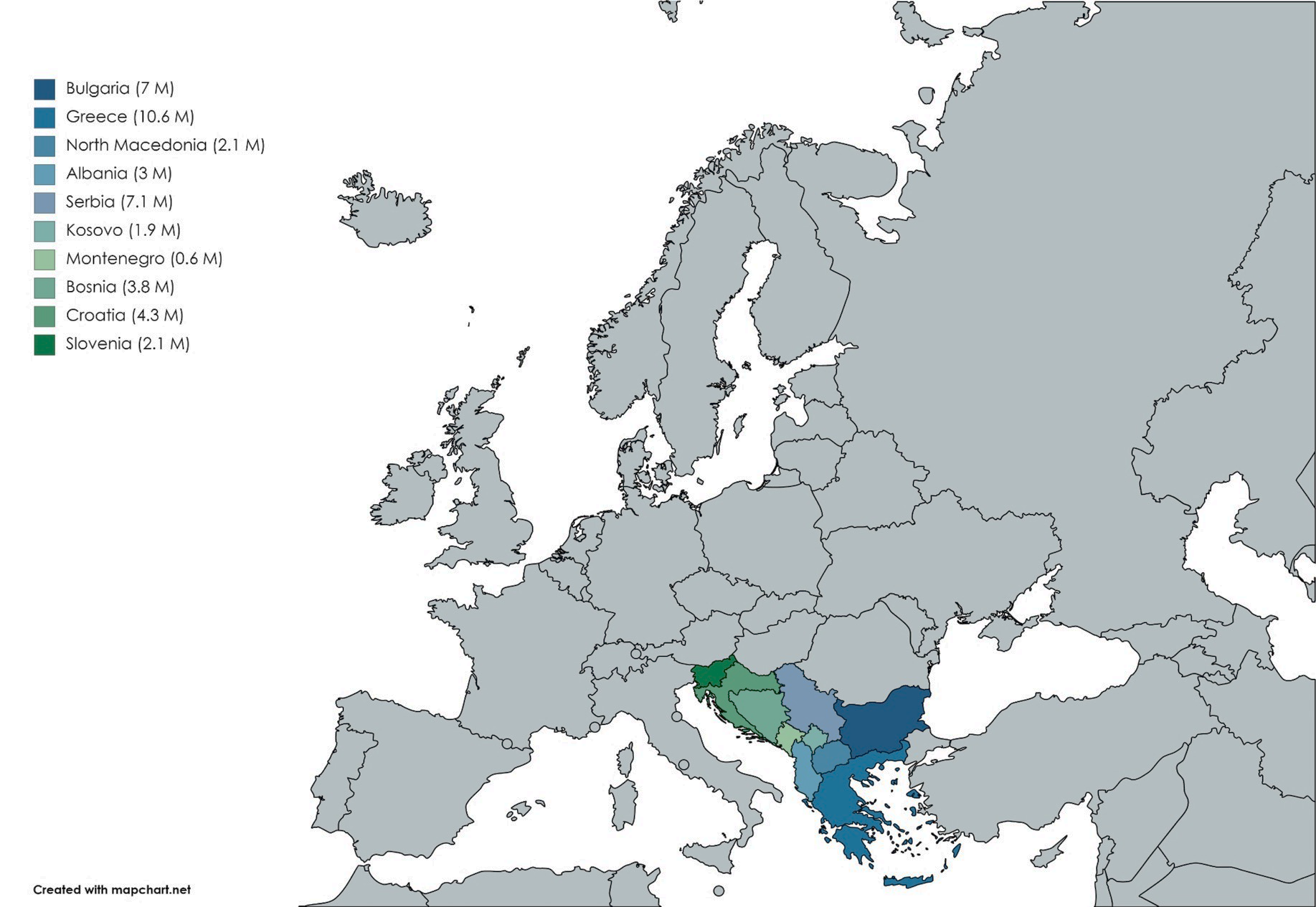


**ANNEX 2**

Top 10 cancers requiring radiotherapy in SEE region (except Kosovo). The ranking was made by absolute numbers of incidence in each of the SEE countries: (*left*) and top 10 in SEE region as a whole (*right*). The table on the right should be used as a legend for the table on the left. It appears that lung, breast and colorectal cancers are the most common in the region, as is the case for Europe as a whole.

| Rank | AL | BH | BG | CRO | GR | MN | MK | SR | SLO |  | Cancer site | # in SEE |  | | |  |  |  |  |  |  |
| --- | --- | --- | --- | --- | --- | --- | --- | --- | --- | --- | --- | --- | --- | --- | --- | --- | --- | --- | --- | --- | --- |
| #1 |  |  |  |  |  |  |  |  |  |  | Lung | 31.783 |  | | |  |  |  |  |  |  |
| #2 |  |  |  |  |  |  |  |  |  |  | Colorectum | 26.872 |  | | |  |  |  |  |  |  |
| #3 |  |  |  |  |  |  |  |  |  |  | Breast | 25.571 |  | | |  |  |  |  |  |  |
| #4 |  |  |  |  |  |  |  |  |  |  | Prostate | 20.498 |  | | |  |  |  |  |  |  |
| #5 |  |  |  |  |  |  |  |  |  |  | Bladder | 14.091 |  | | |  |  |  |  |  |  |
| #6 |  |  |  |  |  |  |  |  |  |  | Stomach | 7.552 |  | | |  |  |  |  |  |  |
| #7 |  |  |  |  |  |  |  |  |  |  | Pancreas | 7.406 |  | | |  |  |  |  |  |  |
| #8 |  |  |  |  |  |  |  |  |  |  | Kidney | 6.213 |  | | |  |  |  |  |  |  |
| #9 |  |  |  |  |  |  |  |  |  |  | Liver | 5.128 |  | | |  |  |  |  |  |  |
| #10 |  |  |  |  |  |  |  |  |  |  | Brain, CNS | 4.979 |  | | |  |  |  |  |  |  |
| AL- Albania, BH – Bosnia-Herzegovina, BG-Bulgaria, CRO-Croatia, GR-Greece, MN-Montenegro, MK-North Macedonia, SR- Serbia, SLO- Slovenia | | | | | | | | | |  | Other sites | 72,222 |  |  |  | |  |  |  |  | 72.222 |
|  |  |  |  |  |  |  |  |  |  |  | Top10 | 150.093 |  |  |  |  |  |  |  |  |  |
|  |  |  |  |  |  |  |  |  |  |  | All Cancers | **222.315** |  |  |  |  |  |  |  |  |  |
